# Investigating conspiracy theories in the light of narrative persuasion

**DOI:** 10.3389/fpsyg.2023.1288125

**Published:** 2023-11-08

**Authors:** Ines Adornetti

**Affiliations:** Cosmic Lab, Department of Philosophy, Communication and Performing Arts, Roma Tre University, Rome, Italy

**Keywords:** conspiracy beliefs, conspiracy theories, narrative, narrative persuasion, transportation

## 1. Introduction

Conspiracy theories (CTs) are beliefs that “two or more actors have coordinated in secret to achieve an outcome, and that their conspiracy is of public interest, but not public knowledge” (Douglas and Sutton, 2023, p. 287). CTs report malicious activities, usually ascribing agency (i.e., enormous capability) to an individual or a group, and are set up in opposition to publicly accepted versions of events. In recent years, the study of CTs has become an important field of research in the psychological sciences (e.g., Swami et al., 2014; Brotherton, 2015; Douglas et al., 2017; van Prooijen and van Vugt, 2018; Georgiou et al., 2019; van Prooijen, 2022; Pilch et al., 2023; van Der Linden et al., 2023). Investigations have focused on proximate mechanisms, such as some cognitive bias (e.g., Brotherton and French, 2015; Wagner-Egger et al., 2018), and the distal evolutionary origins underlying belief in CTs (e.g., van Prooijen and van Vugt, 2018). For example, it has been shown that when people are biased toward inferring intentional explanations for ambiguous actions, they are more likely to endorse CTs (Brotherton and French, 2015), according to which some relevant events are the product of intentional (malevolent) agency rather than ascribable to impersonal forces. Evolutionary psychological models suggest that such cognitive bias is the result of a hyperactive agency detection device, i.e., a human-evolved cognitive system attributing agency to stimuli perceived as intentional, in order to discover potential threats to the individual (Barrett, 2004). From this view, “hyperactive agency detection may facilitate conspiracy thinking as a non-functional consequence” (van Prooijen and van Vugt, 2018, p. 773).

Other lines of research have focused on psychological motives, which Douglas et al. (2017) characterize as epistemic, existential, and social, that may lead people to endorse CTs. Epistemic motives include the need for causal explanations to satisfy the desire for understanding (Van Der Wal et al., 2018). Existential motives relate to compensatory satisfactions: people may be drawn to CTs when they feel unable to exert control over their environment (Goertzel, 1994; van Prooijen and Acker, 2015). Social motives relate to the desire to preserve a positive image of the self or group: CTs exalt the self and the in-group as qualified and moral but as sabotaged by powerful and malevolent others. From this view, people may adhere to CTs defensively, i.e., “to relieve the sense of self or in-group from a sense of culpability for their disadvantaged position” (Douglas et al., 2017, p. 540). Supporting this view, studies have shown that CTs are associated with individual and collective narcissism (Cichocka et al., 2016, 2022) and paranoid ideation (Darwin et al., 2011; Brotherton and Eser, 2015).^1^

In this article, my aim is to contribute to this field of research by highlighting the potential relevance of another factor that has not been explored systematically in the literature so far, i.e., the fact that CTs are great narratives: “[CTs] are easy ways of telling complicated stories. Officially conspiracy theorists tell one story about an event.” (Olmsted, 2011, p. 6). I suggest that insights coming from the field of narrative persuasion (NP) could fruitfully inform the study of the psychological factors accounting for the endorsement of CTs.

## 2. Mechanisms of narrative persuasion

In the context of persuasion, narrative has for a long time been regarded in opposition to argumentation. As pointed out by Bilandzic and Busselle (2013), this opposition can be traced back to Aristotle's distinction between *logos*—the domain of logic and reason—and *pathos*—the domain of emotion, poetry, and stories. Therefore, over time, persuasion has become synonymous with argument—the putting forth of claims and supporting evidence linked by rational or logical coherence. Conversely, narrative—a set of temporally and causally connected sequences of events, determined by the goals and motives of one or more characters, which unfold toward a conclusion (Adornetti et al., 2022, 2023) providing resolution to unanswered questions or unresolved conflicts (Hinyard and Kreuter, 2007, p. 778)—was conceived as a mere description of events with the aim of entertaining. In the last decades, this dichotomy has been challenged. It has been shown that, like argumentation, narrative is a powerful tool of persuasion as it activates some mechanisms that turn out to be particularly effective to modify or change people's beliefs and attitudes (e.g., Mazzocco et al., 2010; Shen et al., 2014; Braddock and Dillard, 2016; Gustafson et al., 2020; Ferretti, 2022; Glaser and Reisinger, 2022).

The new way of conceiving narrative in the light of persuasion has been strongly influenced by the transportation theory advanced by Gerrig (1993) and further expanded by Green and Brock (2000, 2002) with the Transportation-Imagery Model (TIM). According to the TIM, in narrative processing, the dominant experience is *transportation* into the story plot, i.e., a state of intense cognitive and emotional focus on the story that prevents or reduces counterarguing: “while the person is immersed in the story, he or she may be less aware of the real-world facts that contradict assertions made in the narrative” (Green and Brock, 2002, p. 703). Losing access to some real-world facts, when immersed in a narrative, people are thus more prone to accept the story world that the author has created, being less likely to counterargue what the story asserts (see also Busselle and Bilandzic, 2009). According to another important theoretical model, the Extended Elaboration Likelihood Model (Slater and Rouner, 2002), transportation (or involvement) in the narrative is not the only mechanism accounting for NP: a crucial role is also played by *identification* with the story's character (usually mediated by emotional processes). This mechanism leads the audience to adopt the perspective of a character and see the narrated events through the character's eyes, thus embracing their beliefs and way of thinking (e.g., de Graaf et al., 2012; Murphy et al., 2013).

Such a theoretical framework generates predictions about the specific effects of NP and about the correlations between those effects. For example, some studies comparing narratives and non-narratives found that the former are more persuasive (e.g., Murphy et al., 2013; Bullock et al., 2021). Moreover, it has been shown that the more people are transported in a narrative, the more they tend to endorse the beliefs advocated in that story (e.g., Green and Brock, 2002; Dal Cin et al., 2004; Wang and Calder, 2006). Research has also found that the more the audience identifies with a story's character, the more it tends to adhere to their belief system, taking on the character's goals and plans (de Graaf et al., 2012; Hoeken et al., 2016; Igartua and Casanova, 2016). This is especially true in advertising, in which characters extol the virtues of a product in the format of a drama. As demonstrated by Deighton et al. (1989), stories in television commercials are processed empathically; thus, viewers are less disposed to argue and tend to accept the commercial's story world.

## 3. Conspiracy theories and narrative persuasion

Although the narrative format of CTs is widely recognized (e.g., Brotherton, 2015), surprisingly, the relevance of NP in investigating CTs has not been acknowledged as much as it deserves. To the best of my knowledge, only one study has explicitly referred to the theoretical framework of NP to explore the endorsement of CTs (Nera et al., 2018). Other investigations studied the mere exposure to conspiracy narratives, e.g., the film *JKF* about the assassination of President John F. Kennedy (Butler et al., 1995), or the usefulness of narrative intervention to counterargue and reduce conspiracy beliefs (CBs) (e.g., Lazić and ŽeŽelj, 2021; Biddlestone et al., 2023). I will briefly discuss the study by Nera et al. (2018) since it is the only research conducted on the topic. My aim is to highlight some methodological limitations of this research so that future research could take these into account.

Nera et al. (2018) conducted two studies in which they exposed four groups of participants (two for each study) to an X-Files episode in which the protagonists discover a global conspiracy. In both studies,^2^ participants of the experimental condition filled in a questionnaire measuring the endorsement of CBs after viewing the episode; in the control condition, participants responded to the same questionnaire before the viewing. The authors hypothesized that people in the experimental condition would show greater endorsement of CBs; however, the results did not confirm this hypothesis. Moreover, an unexpected effect was found: the control group (in Study 1) reported greater accord with two CBs related to the episode than the experimental group did. According to these results, narrative does not have a persuasive effect, i.e., a role in the endorsement of CTs.

In my opinion, the results by Nera et al. (2018) are affected by some methodological weaknesses. The authors used a design similar to those of previous studies (e.g., Butler et al., 1995; Igartua and Barrios, 2012), comparing two groups exposed to the same narrative condition, with the only difference being that the experimental group answers the questionnaire about CBs after viewing the episode, while the control group does it before. Therefore, this design does not measure a possible *change* of beliefs about CTs in a single group of participants; it focuses on the differences between two different groups regarding their CBs. It is implicitly assumed that the two groups are homogeneous in their prior knowledge and belief systems. This is a relevant point because, as Nera et al. (2018, p. 7) recognize, an audience's prior knowledge about the topics developed in the story influences NP (e.g., Green and Brock, 2000). Only if (before the experiment) the groups are homogenous in their beliefs about the narrative's theme can it be reasonably assumed that comparing their beliefs (measured in one group after the exposure to the content and in the other before) might provide reliable data about the persuasive effect of a message. In the study by Nera et al. (2018), the prior beliefs of the groups about CTs were not investigated. The cognitive style of the groups was not explored as well (see on this point Gjoneska, 2021). Thus, it cannot be excluded that the experimental groups tended to adopt cognitive styles (e.g, reasoning strategies) decreasing conspiracy mentality more than the control groups such as, for example, analytic thinking, which has been shown to reduce belief in CTs (Swami et al., 2014). Indeed, in Study 1 (Nera et al., 2018) the control group, which answered the questionnaire *before* viewing the episode, “showed slightly greater agreement with two conspiracy beliefs associated with the episode than did the experimental group” (p. 5). This result opens to the possibility that, at the start, the control group was more conspiratorial than the experimental group. It cannot be excluded that this affected the results showing that narrative does not have a persuasive effect. Therefore, it is important, when adopting this methodology, to control for these variables and other aspects linked to topics developed in the story.

Another design that could be employed to evaluate CTs in the light of NP might focus on evaluating a possible change in participants' belief systems. To this aim, CBs of both the experimental and control groups should be measured twice: at T0 before exposure to a conspiracy narrative (the experimental group) and a non-conspiracy narrative (the control group) and at T1 after the exposure. Provided that the two groups are homogeneous in their prior beliefs in CTs, if a difference in beliefs can be observed at T1 compared to T0 only in the experimental group, it can reasonably be assumed that this change occurred because of the exposure to the conspiracy narrative. Several studies on narrative persuasion have employed this design, with some investigations finding a persuasive effect of the stories, i.e., a change in participants' beliefs (e.g., Gustafson et al., 2020) before and after the exposure to a narrative message. This point is crucial. Since persuasion is an act to “modify/change the beliefs and attitudes of other people in order to make the recipient act in a certain way” (Ferretti and Adornetti, 2021, p. 2), in my opinion, a design of this kind, allowing the researchers to investigate a possible change in the participants' beliefs system (more directly than the design used by Nera et al., 2018), could provide useful tools to investigate CTs in the light of NP.

## Author contributions

IA: Conceptualization, Methodology, Writing – original draft, Writing – review & editing.

## Footnotes

^1^Political, demographic, and contextual factors involved in the endorsement of CTs have also been investigated (e.g., Claassen and Ensley, 2016; Freeman and Bentall, 2017).

^2^The designs of the two studies are quite similar; some differences relate to the number of variables, which was higher in Study 2, and the duration of the episode, which was longer in Study 1.
